# Sex Differences in Depression and Anxiety Symptoms: Measurement Invariance, Prevalence, and Symptom Heterogeneity Among University Students in South Africa

**DOI:** 10.3389/fpsyg.2022.873292

**Published:** 2022-05-31

**Authors:** N. Florence Tadi, Kaylene Pillay, Ufuoma P. Ejoke, Itumeleng P. Khumalo

**Affiliations:** ^1^Department of Psychology, University of the Free State, Bloemfontein, South Africa; ^2^Department of Psychology, University of Johannesburg, Johannesburg, South Africa

**Keywords:** anxiety, depression, measurement, sex, Africa, measurement invariance, latent class analysis

## Abstract

Adequate measurement is an essential component of the assessment of mental health disorders and symptoms such as depression and anxiety. The present study investigated sex-specific differences in the Patient Health Questionnaire-9 (PHQ-9) and Generalized Anxiety Disorder-7 (GAD-7). This comprehensive cross-sectional design study pursued four objectives: measurement invariance of PHQ-9 and GAD-7 between male and female; depression and anxiety prevalence differences; cross-sex differences in the relationship between depression and anxiety; and a comparison of symptom heterogeneity. A sample of 1966 (male = 592; female = 1374; mean age = 21 years) students from South Africa completed the PHQ-9 and the GAD-7. Data analyses for measurement invariance, latent class analysis, inter-variable correlations and group comparisons were conducted in Mplus. The two-dimensional PHQ-9 achieved scalar invariance, while the GAD-7 yielded metric invariance. The somatic and non-somatic latent dimensions of depression were compared and showed no significant difference between male and female groups. The positive relationship between depression and anxiety was also not significantly different between the two groups. While the PHQ-9 symptoms formed three classes in the male group, and four classes in the female group, the GAD-7 had the same number of classes (three) and a similar pattern between the two groups. These findings hold implications for the measurement, assessment and understanding of symptom manifestation and distribution, as well as the treatment of depression and anxiety in South Africa.

## Introduction

The high prevalence of depression and anxiety is a known and generally accepted reality worldwide, affecting countries ranging from the African region to Euro-American settings, and across degrees of wealth capital (Chisholm et al., 2016; Alonso et al., 2018; Charlson et al., 2019). This is such a universal phenomenon that Scorza et al.’s (2018) study concluded that there were fewer differences in cross-national prevalence than previously thought. Not only is the experience of depression and/or anxiety responsible for major disability in the affected individuals (Whiteford et al., 2013), but it is also detrimental to the economies of the world, and poses a significant disease burden on national healthcare systems (Chisholm et al., 2016). The high disease burden accounted for by mental disorders and other neurological and substance-use disorders bears consequences for government budgets and would overwhelm the systems of mental healthcare (Whiteford et al., 2015; Chisholm et al., 2016).

More specifically, depression is the second largest contributor to the non-fatal disease burden globally (Scorza et al., 2018), while generalized anxiety is the most commonly presenting form of anxiety pathology in the general population (Spitzer et al., 2006). Most research and intervention efforts toward understanding, preventing and treating depression and anxiety rely on accurate and dependable assessment and measurement procedures. Thus, the present study makes the case for the acute need for adequate sex-specific measurement properties of depression and anxiety in a South African context (see Makhubela, 2021).

Mental disorders, especially depression and anxiety, affect a significant proportion of the South African population, forming an upward trend that is expected to increase (Van der Walt et al., 2020; Rousseau et al., 2021). South Africa is categorized as an upper-middle income country (Evans-Lacko et al., 2018; Scorza et al., 2018), characterized by many social ills, including poverty, income inequality and high unemployment, which affect the mental health of its citizens. Except for a limited number of studies, not the least being those of Makhubela (2016, 2021), Makhubela and Debusho (2016), Schneider et al. (2015) and Makhubela and Khumalo (2022), there is insufficient research on the dynamics of depression and anxiety assessment in South Africa. Nonetheless, the contribution of these studies is worth noting. Makhubela (2021) used network analysis to investigate the interaction of depressive and anxiety symptoms, and their gender differences, finding no significant gender differences in the depression networks. Mosotho et al.’s (2008) inquiry into depression among Sesotho-speaking people in South Africa found that depression mainly presents in the form of somatic symptoms, perceptual disturbances and disturbances of the thought processes. As it is clear from the reviewed literature, the question of how sex accounts for depression and anxiety assessment, using the Patient Health Questionnaire-9 (PHQ-9) and the Generalized Anxiety Disorder-7 (GAD-7) in South Africa, remains to be explored.

Based on the socio-environmental factors, such as poverty, civil unrest, cultural diversity and sex inequality, which characterize the landscape of developing countries, it may be plausible to ascribe higher rates of mental disorders and their disease burden to female rather than male persons (Patel et al., 2001). Both gender and sex disproportionately place women at greater exposure to mental disorders (Riecher-Rössler, 2017; Keng et al., 2019). International literature on psychiatric epidemiology has been consistent in reporting sex and gender differences in mental disorders (McLean et al., 2011; Riecher-Rössler, 2017). The gender gap in mental disorders, specifically depression (Kuehner, 2017) and anxiety (Li and Graham, 2017), shows that women are more likely to develop depression and/or anxiety than men. As much as there may be some international data (e.g., Kuehner, 2017; Li and Graham, 2017) indicating the causes of gender and sex differences, such determinations are not so clear in certain contexts like South Africa. In this regard, Makhubela (2016) has opined that the reasons for gender differences are not yet understood. However, in addition to the biological, cultural, environmental and regional/national factors, an often neglected potential reason for group differences in the performance of measuring instruments is non-invariance (Scorza et al., 2018). Although the overlap between biologically assigned/identifying sex and the socially constructed gender is recognized, the present study is concerned with the variable of sex as male and female. Thus, gender and sex are not to be used nor interpreted in an interchangeable fashion.

For this study, we chose, for several reasons, the PHQ-9; (Kroenke et al., 2001; Kroenke and Spitzer, 2002) and the Generalized Anxiety Disorder-7 (GAD-7; Spitzer et al., 2006) as measures for depression and anxiety, respectively. The PHQ-9 and GAD-7 are widely used measures for screening (Teymoori et al., 2020a,b; Borgogna et al., 2021). Their items correspond with the DSM assigned symptoms for depression and generalized anxiety, respectively (Kroenke et al., 2001; Kroenke and Spitzer, 2002; Spitzer et al., 2006; Baas et al., 2011). They were developed as brief self-report screening tools which could be applied in both the general population and clinical settings. Many studies across the world have demonstrated the psychometric properties of the PHQ-9, and have either confirmed a unidimensional structure (e.g., Baas et al., 2011; Bhana et al., 2015; Galenkamp et al., 2017) or a two-dimensional structure of depression categorized into somatic symptoms and non-somatic symptoms (Kendel et al., 2010; Elhai et al., 2012; Forkmann et al., 2013; Beard et al., 2016; Makhubela and Khumalo, 2022). The present study operationalizes depression as comprising somatic and non-somatic depression components. On the other hand, based on the DSM stipulated symptoms of generalized anxiety, the GAD-7 was designed and developed as a brief self-report scale to identify probable cases of GAD (Spitzer et al., 2006). The unidimensional model of the anxiety measure, also used in the present study, has been found to be psychometrically sound (Teymoori et al., 2020b).

Beyond intragroup variations in the determinants and outcomes of depression and anxiety, it is also important to evaluate the intergroup mean comparisons. Such evaluations require measurement invariance, without which the claimed group differences in the scores of measured latent constructs become misleading and without meaning (Cheung and Rensvold, 2002; Chen, 2008; Baas et al., 2011). Measurement invariance, which refers to “the equivalence of a measured construct in two or more groups” (Chen, 2008, p. 1005), assumes that when scale scores are compared, the measuring instrument used measures the same construct across the groups of interest (Chen, 2008). When referring to cross-national comparisons, Scorza et al. (2018) have warned that some of the differences may be a function of non-invariance. To ascertain whether scores on the PHQ-9 and GAD-7 were a function of gender-dependent responding, Borgogna et al. (2021) tested their measurement invariance across 16 gender and sexual minority groups. They found that only cisgender men and women evidenced residual invariance on the PHQ-9; while for the GAD-7, scalar or partial scalar invariance was achieved for most groups (Borgogna et al., 2021). In South Africa, Makhubela (2016) found the Beck Depression Inventory to have scalar invariance, except for two non-invariant items, across race.

The group-specific distribution of symptoms, especially the natural clustering, is a relatively neglected area in the measurement and assessment of mental health. Latent class analysis (LCA) is a person-centered multivariate analysis approach used to elicit naturally occurring unobserved (latent) groupings of participants based on their similarity of categorical endorsement of indicator variables (Rosato and Baer, 2012; Skidmore et al., 2018). Thus, it “uses indicator variables to identify underlying classes of persons in a population” (Skidmore et al., 2018, p. 1246). One of the reasons advanced for the value and importance of performing person-centered analyses such as LCA is their ability to explore atypical presentations and clustering of symptoms as indicator variables (Sullivan et al., 1998; Moreno and Andrade, 2010). Applied to research questions relating to sex differences, LCA has shown significant utility. Whalen et al. (2016) used LCA to study the longitudinal trajectories of depressive symptoms in boys and girls, and found different patterns between the groups. While in girls, high depressive severity remained stable over time, in boys it showed a pattern of fluctuation. In another study, Ploubidis et al. (2007) used LCA to study responses to the General Health Questionnaire (GHQ) and arrived at three latent classes for men and women. While the GHQ indicator variables of self-confidence, perception of work-life, and opportunities to share feelings distinguished the latent groups among women; among the men the variables of work satisfaction and opportunities to share feelings highlighted the differences across groups (Ploubidis et al., 2007).

A vast array of literature across many fields concerned with mental health has continued to be concerned with the issue of the prevalence and nature of the presentation of depression and anxiety symptoms between the sexes. Thus, it is important to explicate the concepts of *gender* and *sex*, as well as their difference, overlap and interaction (Phillips, 2005; Afifi, 2007; Regitz-Zagrosek, 2012). While sex refers to biological indicators, gender is an expression of social factors such as powerlessness, access to resources and constrained roles, thus making gender a social rather than a biological construct (Phillips, 2005). The question of whether differences in the measurement of depression and anxiety are a function of gender and sex is important because it may inform decisions regarding measurement in gender-specific (mental) healthcare (Regitz-Zagrosek, 2012). Research has observed the gender gap in a number of psychological phenomena and circumstances of psychological adjustment. Examples include school engagement and teacher support for autonomy between boys and girls in Belgium (Lietaert et al., 2015); acculturation and the contribution of acculturative stress to depression among Latino college students in universities in the United States (Castillo et al., 2015); and in India, Sharma and Kirmani (2013) found reports of more depression and anxiety symptoms among female than male college students.

The present study applied assessment, measurement and statistical data analysis to explore the psychometric functioning of the measures of depression (PHQ-9) and anxiety (GAD-7) to understand sex differences in the symptom manifestation of the two disorders. In other words, we employed a comprehensive psychometric measurement approach to evaluate how, if at all, sex show differences in the measurement of depression and anxiety symptoms in a group of university students in South Africa. The study focused on equivalence, symptom heterogeneity, the relationship between the two conditions, and a comparison of symptom prevalence between male and female individuals. This aim and focus were underpinned by four specific objectives. The first objective was to investigate the measurement invariance of the PHQ-9 and the GAD-7 separately between the two groups. Following a result of measurement invariance, the second objective was to investigate the depression and anxiety mean score differences between males and females. In the third objective, the strength and direction of the association between depression and anxiety were compared for males and females. The fourth objective was concerned with investigating the homogeneous grouping of symptoms, using LCA, for each group and then comparing emergent profiles between them. Achievement of these objectives presents several contributions to the body of knowledge and practice. Sex-specific measurement, symptom presentation and distribution, and the correct recognition and identification of symptoms (Thai Quynh-Chi et al., 2021) are some of the areas of benefit. The inquiry findings could be useful to policymakers in informing their decisions regarding investment in mental health interventions (see Scorza et al., 2018) in South Africa.

## Materials and Methods

### Participants and Setting

An online cross-sectional survey was conducted among university students, through convenience sampling at a university in South Africa. The online data collection exercise ran for 2 days, and was stopped when the response rate had significantly slowed down. Although 2070 students returned the online questionnaires, only 1988 completed the PHQ-9 and GAD-7 responses in full and were included in this study. Based on frequency analysis, no specific response pattern was observed between the complete and included, and the incomplete and excluded, cases. A further 22 participants who did not indicate their gender/sex were excluded, thus resulting in a sample of 1966 participants. Of these, 592 were male (29.8%; average age 21.67, *SD* = 2.986; ranging from 17 to 44) and 1374 were female (69.1%; average age 21.34, *SD* = 3.150; ranging from 17 to 64).

### Measuring Instruments

#### The Patient Health Questionnaire-9

**FIGURE 1:**
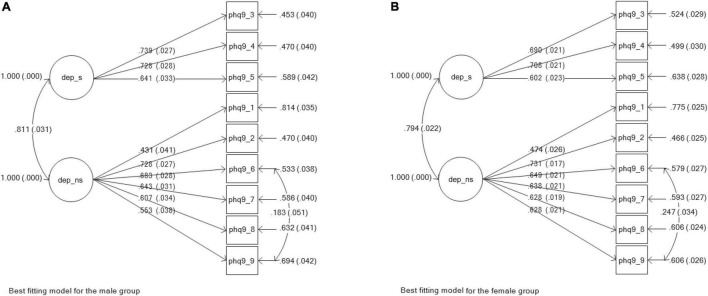
Best fitting models for the measuring instrument of depression (PHO-9). **(A)** Best fitting model for the male group. **(B)** Best fitting model for the female group.

The Patient Health Questionnaire-9 (Kroenke et al., 2001; Kroenke and Spitzer, 2002). The PHQ-9 was designed as an individual level screening tool to determine the presence and severity of depressive symptoms through the frequency of occurrence in the preceding two-week period. We used the self-report computer-based version. As a unidimensional measure, the PHQ-9 has been found to have good construct validity and reliability (e.g., Kroenke et al., 2001; Monahan et al., 2009; Baas et al., 2011; Bhana et al., 2015). In the original study, Kroenke et al. (2001) reported Cronbach’s alpha coefficients of between 0.86 and 0.89. In Kenya, Monahan et al. (2009) found reliability alpha coefficient of 0.78. In a South African sample of chronic care patients, Bhana et al. (2015) found a Cronbach’s alpha of 0.76 for the PHQ-9. Some studies have explored the construct validity of the PHQ-9 without assuming unidimensionality. For example, Elhai et al. (2012) found somatic and non-somatic factors. Galenkamp et al. (2017) also found good fit regarding this two-factor model. In a recent sub-Saharan study, Makhubela and Khumalo (2022) found the PHQ-9 to be reliable, valid, and invariant in and across three countries, namely South Africa, Kenya, and Ghana. In the present study, the measurement model consisting of two factors yielded a better fit (CFI = 0.968; TLI = 0.958). As seen in Figure 1, this model was improved by correlating the residual errors of items 6 (thoughts of self as failure) and 9 (suicide thoughts), resulting in a modification index (MI) of 63.081, and an expected parameter change (EPC) of 0.168. The somatic factor (3 items) had an omega reliability index of 0.730, while the non-somatic factor (6 items) had one of 0.800.

#### Generalized Anxiety Disorder Scale

Generalized Anxiety Disorder scale (Spitzer et al., 2006). The GAD-7 was designed as a brief self-report scale to identify generalized anxiety and assess the severity of symptoms in individuals over the preceding two-week period. Its development and initial validation took place among primary care clinic patients in the US. From this original study, Spitzer et al. (2006) found good validity and reliability. They reported good construct, convergent and divergent validity, as well as a Cronbach’s alpha reliability coefficient of 0.92 and a test-retest interclass correlation of 0.83. Henn and Morgan (2019) validated the GAD-7 in a non-clinical sample of employees in South Africa. They found a good model fit for a one-factor model and reported evidence of discriminant and convergent validity. They also reported a high Cronbach’s alpha coefficient of 0.92. In the present study, the originally intended unidimensional measurement model yielded good fit (CFI = 0.950; TLI = 0.926) and a reliability index of 0.892.

### Procedure and Ethical Aspects

Data were collected online from participants who were all above the age of 18 years. The research process was governed by the research ethics principles as provided in the Declaration of Helsinki (World Medical Association [WMA], 2013) and by the South African Department of Health [DOH] (2014, 2015). This included voluntary participation, confidentiality and anonymity, and post-research care. The Research Ethics Committee of the University of the Free State provided ethical clearance (UFS-HSD2019/1941/2407).

### Data Analysis

#### Construct Validity and Reliability

The omega reliability coefficient was used to compute Structural Equation Modelling (SEM)-based reliability estimates (Raykov, 2004, 2009) of the best fitting measurement models, thus considering the possibility that items could have had correlated errors, as well as unequal factor loadings, in addition to having varying degrees of contribution to the latent factor (Gu et al., 2013). Construct validity was investigated by estimating and finding the best fitting measurement models for the PHQ-9 and GAD-7 for the whole group and for males and females separately. We used the robust maximum likelihood (MLR) estimation (Kline, 2011) in Mplus (version 8.1; Muthén and Muthén, 1998/2017). We used the guidelines stipulated by Hu and Bentler (1999) and recommended by Kline (2011), Byrne (2012) among others. Good fit was therefore shown by smaller and insignificant chi square (χ^2^), a root mean square error of approximation (RMSEA) and a standardized root mean square residual (SRMR) of less than 0.06; a comparative fit index (CFI) of more than 0.95; a Tucker-Lewis index (TLI) of more than 0.95; a smaller Akaike information criterion (AIC) and a smaller Bayesian information criterion (BIC).

#### Measurement Invariance

After establishing the separate well-fitting baseline models, the one-step procedure using the “model is configural scalar metric” Mplus command was implemented (Byrne, 2012; Van de Schoot et al., 2012; Wang and Wang, 2012). This step involved testing for configural, metric and scalar invariance for the two gender groups using a uniform model. The following fit indices were used to examine the fit of the models: the absolute fit indexes – chi-square (χ^2^), AIC and BIC; and the comparative fit indexes – CFI, TLI, RMSEA and SRMR. Good fit is indicated by a higher BIC and AIC; a CFI and TLI lower than or equal to 0.90; a 90% confidence interval; an RMSEA of less than 0.06; and an SRMR of less than or equal to 0.08. Two criteria were used for determining invariance, namely the change in chi-square (Δχ^2^) and the change in CFI (ΔCFI). Measurement invariance is expected to be demonstrated by a ΔCFI of not more than 0.01, and a non-significant Δχ^2^ (Cheung and Rensvold, 2002; Byrne and Van de Vijver, 2010). However, Δχ^2^ is known to be sensitive to sample size (Cheung and Rensvold, 2002; Joshanloo et al., 2013) and thus we placed greater emphasis on the interpretation of ΔCFI.

#### Latent Variable Mean Score and Inter-Variable Correlation Comparison

Descriptive statistics, including mean scores, skewness and kurtosis values, were computed for the whole group, and for males and females separately. The Pearson correlation coefficient was used to determine the size and significance of the relationship between the latent variables of depression and anxiety. Both mean scores (in the case of scalar invariance – assumption of equivalent means) and correlations (in the case of metric invariance – assumption of similar item-to-factor loadings) were compared between males and females.

#### Latent Class Analysis

For the male and female groups separately, we conducted LCA to identify and compare the naturally occurring latent profiles of depression and anxiety. The best fitting models were chosen on the basis of BIC, sample size adjusted BIC (SSABIC), AIC being lower, and the likelihood ratio chi-square (LRχ^2^) lower and not significant, but a statistically significant Lo-Mendell-Rubin adjusted likelihood ratio test (LMR-LRT) and Parametric bootstrapped likelihood ratio test (PB-LRT) (Nylund et al., 2007).

## Results

### Measuring Depression and Anxiety: Estimating the Measurement Models

The two-factor model of depression symptoms, as measured using the PHQ-9, was fitted to the data, firstly involving the whole sample and secondly for separate male and female groups. As displayed in Table 1, all three models showed good to excellent fit (whole group: CFI = 0.956; TLI = 0.939; Male: CFI = 0.974; TLI = 0.964; female: CFI = 0.948; TLI = 0.928). Figure 1 illustrates the PHQ-9 model for males (a) and females (b); factor loadings are also displayed in the figure.

**TABLE 1:** Measurement models.

	Measurement model for the PHQ-9									
Model	N	AIC	BIC	χ^2^	*df*	*P*	RMSEA, *p* [90% CI]	CFI	TLI	SRMR
Model for all	1988	47738	47895	231.302	26	<0.001	0.063,0.002 [0.056.071]	0.956	0.939	0.032
Model for Male	592	14155	14278	60.227	26	<0.001	0.047,0.594 [0.032.063]	0.974	0.964	0.030
Model for Female	1374	32971	33117	190.463	26	<0.001	0.058,0.085 [0.048.067]	0.948	0.928	0.035

	**Measurement model for the GAD-7**									
**Model**	**N**	**AIC**	**BIC**	**χ^2^**	***df***	***P***	**RMSEA, *p* [90% CI]**	**CFI**	**TLI**	**SRMR**

Model with all	1988	34335	34452	262.342	14	<0.001	0.094, <0.001 [0.085.105]	0.950	0.926	0.032
Model for Male	592	10605	10698	54.955	14	<0.001	0.070,0.040 [0.051.090]	0.968	0.952	0.030
Model for Female	1374	23213	23323	237.914	14	<0.001	0.108, <0.001 [0.096.120]	0.940	0.910	0.036

*χ^2^ = chi square; df = degrees of freedom; p = probability estimate; CFI = comparative fit index; TLI = Tucker-Lewis index; RMSEA = root mean square error of approximation; AIC = Akaike information criterion; BIC = Bayesian information criterion; SRMR = standard root mean residual.*

**FIGURE 2:**
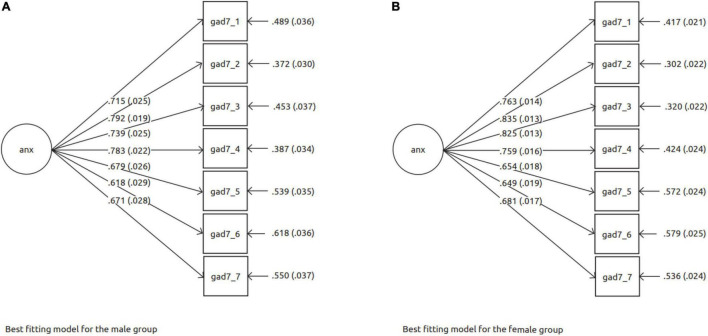
Best fitting models for the measuring instrument of anxiety (GAD-7). **(A)** Best fitting model for the male group. **(B)** Best fitting model for the female group.

Good model fit was also found for the unidimensional model of anxiety symptoms, as measured by the GAD-7 (whole group: CFI = 0.950; TLI = 0.926; male: CFI = 0.968; TLI = 0.952; female: CFI = 0.940; TLI = 0.910). These models formed the baseline models with which we could begin testing for measurement invariance. Figure 2 illustrates the GAD-7 model for males (a) and females (b); factor loadings are also displayed in the figure.

### Measurement Invariance Between the Gender Groups for the Depression and Anxiety Measures

The one-step procedure in Mplus for investigating measurement invariance (Van de Schoot et al., 2012) by testing for configural, metric and scalar invariance was used for the PHQ-9 and GAD-7. As seen in Table 2, the results showed full scalar invariance for the PHQ-9 model, but only metric invariance for the GAD-7 model.

**TABLE 2:** Measurement invariance testing the PHQ-9 and GAD-7: male and female.

Patient Health Questionnaire – 9													
**Model**	**χ^2^**	***df***	***P***	**CFI**	**TLI**	**RMSEA, *p* [90% CI]**	**AIC**	**BIC**	**SRMR**	**Δχ^2^**	***df***	***p***	**ΔCFI**
Configural model	250.258	52	<0.001	0.955	0.938	0.062,0.004 [0.055.070]	47126	47439	0.034	.	.	.	.
Metric model	267.713	59	<0.001	0.953	0.943	0.060,0.012 [0.053.067]	47124	47398	0.037	13.464	7	0.062	0.002
Scalar model	302.560	66	<0.001	0.947	0.942	0.060,0.006 [0.054.067]	47145	47380	0.039	49.744	14	<0.001	0.008

**General Anxiety Disorder – 7 scale**													
**Model**	**χ^2^**	***df***	***P***	**CFI**	**TLI**	**RMSEA, *p* [90% CI]**	**AIC**	**BIC**	**SRMR**	**Δχ^2^**	***df***	***p***	**ΔCFI**

Configural model	280.093	28	<0.001	0.949	0.924	0.096, <0.001 [0.086.106]	33819	34054	0.034	.	.	.	.
Metric model	307.065	34	<0.001	0.945	0.932	0.090, <0.001 [0.081.100]	33813	34014	0.036	7.209	6	0.302	0.004
Scalar model	357.971	40	<0.001	0.936	0.932	0.090, <0.001 [0.082.099]	33850	34018	0.041	62.739	12	<0.001	0.013

*χ^2^ = chi squared; df = degrees of freedom; p = probability estimate; CFI = comparative fit index; TLI = Tucker-Lewis index; RMSEA = root mean square error of approximation; AIC = Akaike information criterion; BIC = Bayesian information criterion; SRMR = standardized root mean residual.*

### Patient Health Questionnaire-9 Measurement Invariance

The configural model was tested between the two groups and showed good fit [χ^2^(52) = 250.26, ρ < 0.001; CFI = 0.955; TLI = 0.938]. The metric model resulting from equality constraints on factor loadings was also well fitting [χ^2^(59) = 267.71, ρ < 0.001; CFI = 0.953; TLI = 0.943]. The ΔCFI of 0.002 indicated full metric invariance. Scalar invariance was also achieved as shown by ΔCFI of 0.008. This model fitted the data well [χ^2^(66) = 302.56, ρ < 0.001; CFI = 0.947; TLI = 0.942].

### Generalized Anxiety Disorder-7 Measurement Invariance

Similar to the PHQ-9 model, the configural model for GAD-7 showed good fit [χ^2^(28) = 280.09, ρ < 0.001; CFI = 0.949; TLI = 0.924]. The GAD model [χ^2^(34) = 307.07, ρ < 0.001; CFI = 0.945; TLI = 0.932] demonstrated metric invariance (ΔCFI = 0.004). However, the scalar model [χ^2^(40) = 357.97, ρ < 0.001; CFI = 0.936; TLI = 0.933] did not show invariance between the groups (ΔCFI = 0.013).

#### Comparison of Mean Scores and Correlations of Depression and Anxiety Between the Sexes

Because the PHQ-9 attained scalar invariance, the dimensions of somatic depression and non-somatic depression could be compared for mean scores. For somatic depression, males scored 4.51 (*SD* = 0.12), while the females scored 5.66 (*SD* = 0.07), which were not significantly different, Wald (*df* = 1) = 3.17, ρ = 0.075. The scores were also not significantly different for the non-somatic dimension, with males scoring 7.23 (*SD* = 0.18) and females scoring 8.28 (*SD* = 0.12), Wald (*df* = 1) = 1.60, ρ = 0.206.

Although the mean scores for anxiety could not be compared because of a lack of GAD-7 scalar invariance, the comparison of the relationship between anxiety and depression scores was allowed on the basis of metric invariance. The standardized correlation between somatic depression and anxiety in the male group was *r* = 0.608 (SE = 0.026), and for the female group it was 0.593 (SE = 0.017), and showed no significant difference, Wald (*df* = 1) = 1.04, ρ = 0.314. For the non-somatic depression and anxiety correlations, the males scored *r* = 0.75 (SE = 0.018), and the females scored *r* = 0.74 (SE = 0.012), with no significant difference, Wald (*df* = 1) = 0.054, ρ = 0.815. Both the means scores and correlations between the groups are displayed in Table 3.

**TABLE 3:** Group comparisons of means and correlations.

Index	Mean scores			
	Depression Somatic		Depression Non-somatic	
	Male	Female	Male	Female
Mean	4.508	5.663	7.225	8.282
Standard error	0.108	0.067	0.178	0.122
Minimum	0	0	0	0
Maximum	9	9	18	18
Median	4	6	7	8
Skewness	0.021	–0.484	0.361	0.202
Kurtosis	–1.115	–0.718	–0.614	–0.852
Wald	3.170		1.601	
Degrees of freedom	1		1	
Probability value	0.0750		0.2057	

**Index**	**Inter-variable correlations**			
	**Depression Somatic and Anxiety**		**Depression Non-somatic and Anxiety**	
	**Male**	**Female**	**Male**	**Female**

Standardized correlation	0.608	0.593	0.751	0.740
Standard error	0.026	0.017	0.018	0.012
Wald test	1.014		0.054	
Degrees of freedom	1		1	
Probability value	0.3139		0.8155	

#### Latent Class Analysis – Depression Symptom Heterogeneity Between the Sexes

In the male group, the model for one-, two-, three- and four-class solutions was estimated, and the model fit indices showed the three-class solution to be the best fitting. A similar procedure was followed, fitting up to a five-class model, for the female group, with the results fielding a best fitting four-class model. As displayed in Table 4, the male three-class solution was characterized by the lowest entropy value, and non-significant LMR-LRT and VLM-LRT, with class 1 accounting for 43.6%, class 2 for 35.3% and class 3 for 21.1%. Informed by both the relative fit indices and parsimony, the four-class solution was chosen for the women. As seen in Table 4, this is characterized by a low entropy value, and non-significant LMR-LRT and VLM-LRT, with the class distribution of 25.5% for class 1, 18.0% for class 2, 32.1% for class 3 and 24.5% for class 4.

**TABLE 4:** Latent class solution model fit indices using the symptoms of depression (PHQ-9) for the male and female groups separately.

Male group (*n* = 592)														
**Model**	**Log likelihood**	**AIC**	**BIC**	**SSA BIC**	**Entropy**	**LMR- LRTρ**	**VLM- LRTρ**	**PB-LRTρ**	**Percentage**				
									**Class 1**	**Class 2**	**Class 3**	**Class 4**	**Class 5**
1 Class	–6977	14009	14127	14042	.	.	.		100	.	.	.	
2 Classes	–6404	12919	13161	12986	0.835	<0.001	<0.001	<0.001	42.6	57.4	.	.	
**3 Classes**	–**6265**	**12696**	**13059**	**12796**	**0.786**	**0.108**	**0.105**	<**0.001**	**43.6**	**35.3**	**21.1**	.	
4 Classes	–6206	12634	13120	12768	0.808	0.529	0.528	<0.001	44.4	27.4	18.6	9.6	

**Female group (*n* = 1374)**													
**Model**	**Log likelihood**	**AIC**	**BIC**	**SSA BIC**	**Entropy**	**LMR- LRTρ**	**VLM- LRTρ**	**PB-LRTρ**	**Percentage**				
									**Class 1**	**Class 2**	**Class 3**	**Class 4**	**Class 5**

1 Class	–16165	32385	32526	32440	.	.	.		100	.	.	.	.
2 Classes	–14842	29795	30082	29908	0.831	<0.001	<0.001	<0.001	44.3	55.7	.	.	.
3 Classes	–14554	29275	29709	29445	0.784	<0.001	<0.001	<0.001	25.1	45.1	29.8	.	.
**4 Classes**	–**14457**	**29137**	**29717**	**29365**	**0.756**	**0.267**	**0.264**	<**0.001**	**25.5**	**18.0**	**32.1**	**24.5**	.
5 Classes	–14372	29023	29750	29308	0.747	0.161	0.159	<0.001	8.2	28.1	19.3	16.1	28.4

**FIGURE 3:**
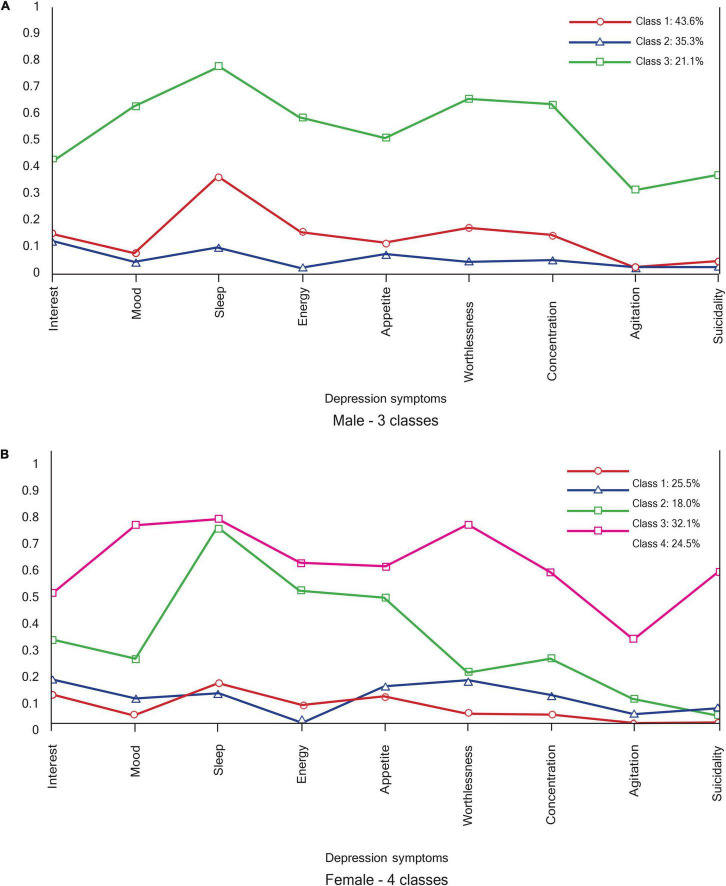
Depression profiles. **(A)** Male – 3 classes. **(B)** Female – 4 classes.

As displayed in Figure 3, the depression class profiles for men and women were interpreted on the basis of the relative scores for each depression symptom. Three depression class profiles emerged in the female group. Class 1 accounting for 43.6% shows high depression, characterized by high sleep disturbance and mood disturbances. Class 2 (35.3%: low) and Class 3 (21.1%: low, with sleep disturbance) both show low depression, with relatively low scores across all symptoms, except for sleep disturbance in Class 3.

Four depression class profiles emerged in the male group, with two high depression profiles (32.1% for class 3 = high, somatic; and 24.5% for class 4 = high) and two low depression profiles (25.5% for class 1 = low, and 18.0% for class 2 = low, cognitive). Although both class 3 and class 4 show high levels of depression symptom endorsement, the class 3 profile (high, somatic) is distinguished by high sleep, energy, and appetite disturbances, while other symptoms are scored lower. Class 2 (low, cognitive) has a higher endorsement of appetite and concentration disturbances and interest, as well as worthlessness.

### Latent Class Analysis – Anxiety Symptom Heterogeneity Between the Sexes

Model fit indices for the male and female anxiety symptom latent profiles are displayed in Table 5. In both groups, the three-class models were the best fitting, as indicated by low entropy value and non-significant LMR-LRT and VLM-LRT, with the four-class models adding no further fit improvement. In the male group, class 1 comprised 25.2%, class 2, 42.2%, and class 3, 32.6% of the sample. In the female group, class 1 accounted for 40.0%, class 2 for 37.0%, and class 3 for 23.0%.

**TABLE 5:** Latent class solution model fit indices using the symptoms of anxiety (GAD-7) for the male and female groups separately.

Male group (*n* = 592)												
**Model**	**Log likelihood**	**AIC**	**BIC**	**SSA BIC**	**Entropy**	**LMR- LRT ρ**	**VLM- LRT ρ**	**PB-LRT ρ**	**Percentage**			
									**Class 1**	**Class 2**	**Class 3**	**Class 4**
1 Class	–5621	11284	11376	11309	.	.	.	.	100	.	.	.
2 Classes	–4958	10003	10191	10055	0.855	<0.000	<0.000	<0.000	50.3	49.7	.	.
**3 Classes**	–**4773**	**9677**	**9962**	**9755**	**0.831**	**0.672**	**0.669**	<**0.000**	**25.2**	**42.2**	**32.6**	.
4 Classes	–4698	9570	9952	9675	0.814	0.752	0.752	<0.000	17.6	20.0	37.4	25.0

**Female group (*n* = 1374)**												
**Model**	**Log likelihood**	**AIC**	**BIC**	**SSA BIC**	**Entropy**	**LMR- LRT ρ**	**VLM- LRT ρ**	**PB-LRT ρ**	**Percentage**			
									**Class 1**	**Class 2**	**Class 3**	**Class 4**

1 Class	–12657	25357	25466	25400	.	.	.	.	100	.	.	.
2 Classes	–10950	21986	22211	22074	0.871	<0.000	<0.000	<0.000	42.0	58.0	.	.
**3 Classes**	–**10445**	**21020**	**21360**	**21153**	**0.848**	<**0.000**	<**0.000**	<**0.000**	**40.0**	**37.0**	**23.0**	.
4 Classes	–10307	20789	21244	20968	0.828	0.128	0.125	<0.000	15.2	35.0	15.0	34.8

**FIGURE 4:**
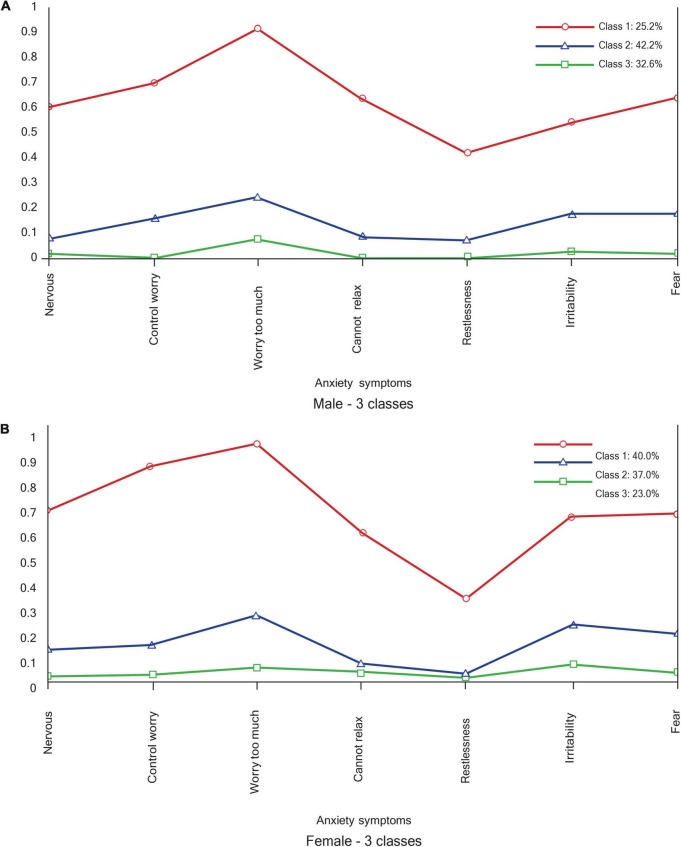
Anxiety profiles. **(A)** Male – 3 classes. **(B)** Female – 3 classes.

As seen in Figure 4, the anxiety class profiles for males and females were interpreted on the basis of the relative scores of each anxiety symptom. The profiles of the two groups do not seem to differ, with both of them having a high anxiety profile (class 1), with high endorsement of worrying too much and having the need to control the worry. Similarly, two low anxiety profiles are observed in both groups (class 2 and class 3). While all of class 2 symptoms are higher than all of class 3 symptoms in the male group, the female group’s poor relaxation and restlessness symptom endorsement overlap.

## Discussion

Our aim was to evaluate the measurement of depression and anxiety by investigating the measurement properties of the PHQ-9 and the GAD-7 among male and female university students in South Africa. For the two measuring instruments, we used the university student data (*N* = 1966) to test for measurement invariance, comparing latent mean scores, naturally occurring symptom homogeneity, and depression and anxiety correlation between men (*n* = 592) and women (*n* = 1374). While we found scalar invariance for the two-factor PHQ-9 (somatic and non-somatic depression), the unidimensional GAD-7 yielded metric invariance. Allowing for mean score comparison of somatic and non-somatic depression, we found similar mean scores with no significant group difference. As expected, depression and anxiety were positively correlated, with no significant difference between men and women. Interestingly, while responses to the anxiety measure yielded three latent classes with similar profiles for the men and women, the LCA results for the depression measure showed different models (number of classes and nature of profiles) for men and women.

### Measurement Invariance for Patient Health Questionnaire-9 and Generalized Anxiety Disorder-7

The two-factor PHQ-9 measure, with distinct somatic and non-somatic depression dimensions, showed scalar invariance across gender, and their mean scores were compared. In both groups, this model had covarying residual errors for items 6 (thoughts of self as failure) and 9 (suicide thoughts). The unidimensional GAD-7 only achieved metric invariance. While the latter instrument shows only the equivalence of indicator factor loadings on the intended latent constructs, the former assumes the equivalence of intercepts between the groups, and thus the means could be compared. In addition to the intercept equivalence (scalar) in intergroup depression measurement, the significance of the similarity of the factor structure (configural) and intended indicators (metric) of somatic and non-somatic dimensions of depression is worth noting. This finding means that in this sample, the structure of depression is distinguished by an evaluation of psychosomatic symptoms (Kendel et al., 2010; Elhai et al., 2012; Forkmann et al., 2013; Beard et al., 2016).

### Comparison of Mean Scores and Inter-Variable Correlations Between Depression and Anxiety

The empirical evidence of no group differences in our data regarding the prevalence and manifestation of depression and anxiety is not supported by previous epidemiological data (e.g., Whiteford et al., 2015; Kuehner, 2017; Riecher-Rössler, 2017). Literature has predominantly found a gender gap, showing women to experience greater depression and anxiety symptoms as well as accounting for a greater disease burden. The empirically supported assertion that “women are about twice as likely as are men to develop depression during their lifetime” (Kuehner, 2017, p. 146) is often made, but such a result did not emerge in this cross-sectional study. It is also known that the gender gap in depression and depressive symptoms varies across countries (Salk et al., 2017). Salk et al.’s (2017) view that the perpetual “emphasizing the preponderance of women with depression is that depression becomes a female-stereotyped disorder” (p. 808), and that depression in men becomes a neglected phenomenon, helped to contextualize our results.

The question of whether gender/sex moderated the relationship between depression and anxiety was pursued through multi-group comparison of the association between their latent variables. We found the positive associations of somatic and non-somatic dimensions of depression with anxiety to be positive and high, with no significant difference between the men and women. While Teymoori et al. (2020a) did not make a distinction between somatic and non-somatic depression, they found a positive relationship between unidimensional PHQ-9 and GAD-7. Given Mosotho et al.’s (2008) findings of predominant somatic presentation among a South African sample, we would have expected this somatic/non-somatic distinction to play a greater role.

### Latent Class Analysis for Patient Health Questionnaire-9

We found different latent class profiles for men (three classes) and women (four classes) with differing patterns. The finding of gender-related subtypes of depression is consistent with findings from previous studies (e.g., Kuehner, 2017; Rudenstine and Espinosa, 2018). Except for the two latent classes of high depression and low depression, which were similar in both groups, two main features distinguished men from women. The first is the high depression with the predominance of somatic symptoms factor, which is reminiscent of what Baumeister and Gordon (2012) refer to as female depression. They ascribe female depression to mood disturbance partly resulting from women’s reproductive system. Depression in women is known to present with atypical symptoms, with the predominance of somatic symptoms such as low energy, fatigue and pain (Kuehner, 2017). In a South African study, Makhubela and Debusho (2016) found women to score higher than men on depression-related somatic complaints.

The second distinguishing feature is low depression with features of cognitive evaluative indicators (depressive cognitions of worthlessness and poor concentration) among the men. The finding of different depression symptom configurations for men and women is a literature-consistent demonstration of the role of gender and sex in the patterns of symptom distribution. When examining depression and anxiety symptoms together in a large population-representative sample in the United States, Rudenstine and Espinosa (2018) found a seven-class model for the males and an eleven-class model for the females.

### Latent Class Analysis for Generalized Anxiety Disorder-7

The latent class models were similar for men and women in terms of both the number of classes and the pattern of symptom endorsement. The patterns were characterized by a predominance of being nervous, worrying too much and a preoccupation with controlling the worry. This similarity between how men and women present with anxiety symptoms is notwithstanding previous studies, which have shown that women were at a higher risk to carry greater disease burden from anxiety diagnoses than men. McLean et al. (2011) found that women were more likely to meet diagnostic criteria for all of the anxiety disorders except for social anxiety disorder, which was the same as men.

### Limitations

We acknowledge the contribution of this manuscript within the context of a few limitations of the study. Data in the present study came from a cross-sectional survey utilizing only self-reported information. Future studies could consider not only implementing longitudinal surveys across time, but also complement self-report information with other forms of data sources. Additional sociodemographic information such as race, gender, sexual orientation and socioeconomic status would have enriched our inquiry. It is known from Makhubela’s (2016) Beck Depression Inventory validation study that, albeit with a couple of non-invariant items, scalar invariance was achieved between black and white participants in South Africa. The present study’s analysis was strictly limited to sex, and not gender. The undisputed distinction between sex (as biological determinants) and gender (as social factors) (see Phillips, 2005; Afifi, 2007; Regitz-Zagrosek, 2012) is acknowledged. Another limitation is that we applied convenience sampling, with available students, instead of a population-based sample. Working with the assumption that depression is a developmental phenomenon, whose prevalence and severity increases as individuals become adults (Black and Pössel, 2015), it may be valuable for future research to explore the measurement complexities of depression and anxiety in a population-based adult sample, and across a lifespan.

### Recommendations and Implications

The findings of the present study have implications for both research and practice, some of which may also be considered by policymakers. They make a case for sex-specific (mental) healthcare for the South African population (see Regitz-Zagrosek, 2012). When the healthcare system is sensitive to sex-imposed impacts, it will also respond to the knowledge that, at present, females account for more disability-adjusted life years (DALYs) in mental and neurological disorders (Whiteford et al., 2015). Such a response would foster progress toward efforts to reduce the treatment gap (see Alonso et al., 2018; Evans-Lacko et al., 2018). This outcome would improve access to treatment and reduce the treatment gap, as currently only a minority of people with mental disorders receive help (Evans-Lacko et al., 2018). Lastly, future research should consider the measurement of depression and anxiety using the recently developed Patient Health Questionnaire – Anxiety and Depression Scale (PHQ-ADS, Kroenke et al., 2019), whose bifactor model explains the combination of the syndrome better (Teymoori et al., 2020a).

## Conclusion

Within a South African context, the present paper was concerned with equivalence, symptom heterogeneity, the relationship between depression and anxiety and a comparison of symptom prevalence between male and female individuals. While depression and anxiety were found to be equally prevalent in both groups, the difference in symptom presentation is noteworthy. Using the GAD-7, we found generalized anxiety symptoms to manifest and present in similar ways between males and females, but the PHQ-9 yielded different depression latent class patterns. These results make a case for adequate sex-specific measurement approaches to depression and anxiety in South Africa.

## Data Availability Statement

The raw data supporting the conclusions of this article will be made available by the authors, without undue reservation.

## Ethics Statement

The studies involving human participants were reviewed and approved by the General/Human Research Ethics Committee University of the Free State. The patients/participants provided their written informed consent to participate in this study.

## Author Contributions

KP collected the data for her Master’s degree research project and was supervised by IK. IK analyzed the data. All authors contributed to conceptualization and writing.

## Conflict of Interest

The authors declare that the research was conducted in the absence of any commercial or financial relationships that could be construed as a potential conflict of interest.

## Publisher’s Note

All claims expressed in this article are solely those of the authors and do not necessarily represent those of their affiliated organizations, or those of the publisher, the editors and the reviewers. Any product that may be evaluated in this article, or claim that may be made by its manufacturer, is not guaranteed or endorsed by the publisher.
